# Tobacco smoking model containing snuffing class

**DOI:** 10.1016/j.heliyon.2023.e20792

**Published:** 2023-10-11

**Authors:** Viswanathan Padmavathi, Kandaswami Alagesan, Samad Noeiaghdam, Unai Fernandez-Gamiz, Manivelu Angayarkanni, Vediyappan Govindan

**Affiliations:** a Department of Mathematics, Thangavel Womens Arts and Science College, Salem - 636 106, Tamil Nadu, India; b Department of Mathematics, Kandaswami Kandar's College, Velur - 638 182, Tamil Nadu, India; c Industrial Mathematics Laboratory, Baikal School of BRICS, Irkutsk National Research Technical University, Irkutsk, 664074, Russia; d Department of Applied Mathematics and Programming, South Ural State University, Lenin prospect 76, Chelyabinsk, 454080, Russia; e Nuclear Engineering and Fluid Mechanics Department, University of the Basque Country UPV/EHU, Nieves Cano 12, 01006, Vitoria-Gasteiz, Spain; f Department of Mathematics, Hindustan Institute of Technology and Science, Chennai, Tamil Nadu, India

**Keywords:** 37M05, 34F05, 92D30, Atangana-Baleanu-Caputo (ABC) derivative, Tobacco smoking model, Epidemic model, q-Homotopy analysis transform method (q-HATM), Mathematical models

## Abstract

In recent years, the world has faced many destructive diseases that have taken many lives across the globe. To resist these diseases, humankind needs medicine to control, cure, and predict the behaviour of such problems. Recently, the Corona virus, which primarily affects the lungs, has threatened the globe. Similarly, tobacco-related illnesses impair the immune system, and this reduces the ability to fight against Covid-19. This tobacco-smoking version is vital for the researchers to reap the solution by using the q-homotopy analysis transform method with the useful resource of the Atangana-Baleanu-Caputo impression. Hence, the graphical illustrations have been discussed to achieve a solution for this mathematical model. This work applies the q-homotopy analysis transform method to the preeminent fractional operator Atangana-Baleanu-Caputo to better comprehend the infectious model of tobacco snuffing and smoking. Figures and tables are used to display the outcomes. The paper also aids in the analysis of the practical theory by predicting how it would behave when compared to the rules when considering the replica. It offers accurate grid point outcomes and fixes. The system's accuracy in the convergent zone is shown by the curves. The smoking model has been illustrated using graphical findings and fractional derivatives for easier comprehension. It's feasible that applications in the real world will make use of fractional derivatives.

## 1 Introduction

People in this world are suffering so much in their lives because of their bad habits. Snuffing Tobacco is one of the dangerous threads. It has been killing more than eight million peoples now and then. Globally seven million people are dying by using tobacco directly. 1.2 million people are affected as passive smoker [4]. Hence tobacco is dangerous, which affects lungs directly and root for many respiratory diseases. World health organisation found a study that smokers are highly affected by Covid-19. They are having high risk factors than non-smokers. The deadly corona virus primarily affects lungs. Smoking impairs lungs, which makes hard to fight against corona. Tobacco is serious threat for cardio vascular and other respiratory diseased people who have severe risk factors when affected by Covid-19 [11], [12], [13], [14].

A study says they might face death constantly WHO evaporating research between tobacco, nicotine and Covid-19. They say smoking or the use of tobacco can make manifestation of Covid-19. Smoking or chewing tobacco directly affects lungs worstly. Tobacco can irritate trachea which leads to breathing problem. Further people with weakened respiratory system are also susceptible to asthma, tuberculosis. The virus Covid-19 mainly affects lungs, if the respiratory system is weak already Covid can be very dangerous [22], [33], [41].

A mathematical model for smoking was developed by Castillo-Garsow et al. in 1997 [11]. The total population was classified into three groups: potential, chain, and permanently stopped smokers, according to the model. Sharomi and Cumel, however, tinkered with the model [42]. Ham surveyed students from various vocational technical institutions in Korea in 2007 to discover the stages and progression of smoking among them [19]. Zaman expanded the prototype by adding a new type of smokers that only smoke on occasion and presenting emphatic communication in a numerical order [47], [48]. For the system to progress to finite time extension, the square root dynamics of a quitting smoking scenario were developed by Zeb et al. [49], [50], [51]. Others provided the smoking model in a fractional and integer order. Snuffing is another method of tobacco use [21], [52]. Till presently, talk about the scientific demonstrate by including the snuffing course, we isolated the whole populace in five classes Susceptibility smokers, snuffing class, irregularity smokers, habitual smokers, and quit smokers seem to be the symbols M(j),L(j),N(j),O(j),P(j) at time *j*, respectively [5], [12], [13], [14].

In last few decades, fractional calculus has been a popular field of study. Fractional calculus has recently been discovered to be valuable in a variety of domains, including quantitative biology, electrochemistry, scatting theory and many other fields [15], [20], [25], [26], [27]. It is also been dubbed the future model for groundwater research [1], [2], [46]. The application of a new numerical method with the ABC operator, which depicts some chaotic phenomena with mathematical equations that can be dealt with the robustness of fractional calculus, has been encouraged in the study [16], [17], [18]. We also provide the q-HATM, which is a novel technique. It is a combination of q-HAM and the Laplace Transform. The flexibility to alter two computational approaches for investigating fractional differential equations is its strength. The convergence point of solution series can be controlled in a huge acceptable domain by choosing correct *h* [35], [37], [38]. More studies and applications of the HAM and HATM can be found in [28], [29], [30], [31].

In the twenty-first century, mathematical biology is a subject that greatly concerns both mathematicians and biologists. Applications in this discipline are numerous. A researcher's primary goal is to use mathematical language to describe the dynamics of infectious illnesses and the various components of their control. The basic framework for mathematical biology was created by [10], who also gave the field a practical foundation. In less than three years, he developed a law regarding the propagation of illness using a probabilistic technique. In contrast to integer-order epidemic models, which have helped us understand biological systems, fractional-order models, particularly in the context of smoking dynamics, present more accurate biological models with memory and after-effect qualities. So, system of derivatives is subjected to fractional-order derivatives. For the fundamentals of fractional calculus and fractional order differential equations, the stability of the system is discussed as being the same as that proved in [6]. Table 1 in [24] provides the biological interpretation of the model's input parameters.

**Table 1:** Parameters and description.

Symbols	Description
*θ*	Recruitment rate (birth or migration)
*χ*_1_	Rate at which susceptible population moves to snuffing class
*χ*_2_	Rate at which snuffing class become irregular smokers
*γ*	Rate at which irregular smokers become regular smokers
*ρ*	Death rate of snuffing class due to tobacco use

In many fields, mathematical modelling can be used to simplify complex real-world problems into straightforward numerical data. By using mathematical modelling techniques, one can predict trends in plant diseases, develop and evaluate preventative measures, prevent global food shortages, and meet one's fundamental nutritional needs. A relevant theory for monitoring a disease's spread is mathematical modelling. Such a model investigates the microorganism's non-faced measurement statistics.

In order to analyse the disease, many models have been constructed. It is possible to analyse the progression of the disease using a variety of procedures. By utilising a variety of numerical and analytical techniques, some researchers have studied the epidemic model of pine wilt and other diseases caused by various viruses. Leibnitz was able to understand a fraction in a derivative, demonstrating how fractional calculus is much more useful for solving current, real-world issues than classical calculus. The most elegant and systematic interpretation of nature's authenticity is provided by fractional calculus. Many fields, including liquid machinery, chemistry, hydrology, ecology, and manufacturing, have significantly improved as a result of the extraordinary applications of fractional differential equations [3], [23], [32].

Alzahrani [6] concerned with a tobacco smoking model that includes a sizeable group of snuff users. In order to achieve this, the formulation of the model incorporating the snuffing class is presented, and the discussion of the equilibrium points for positive and negative smoking follows. The model's local stability is determined by the Hurwitz theorem, while its global stability is determined by Lyaponov function theory. We characterise the ideal level using the Pontryagin maximum concept and several methods for controlling smoking. A nonstandard finite difference (NSFD) scheme and the Runge-Kutta fourth order approach are utilised to solve the suggested model. Lastly, some numerical outcomes for control are shown.

The propagation of finances, seismic waves, permeable mediums, viscoelastic materials and many more physical phenomena are among the many physical processes that the fractional-order differential equations are known to model well. The conventional Mittag-Leffler operation without locality or singularity is the foundation of the kernel ABC fractional derivative. The ABC fractional derivative is a good choice for defining the world's physical and material reality. A more accurate characterisation of memory within a structure at a different level is provided by differences in kernel unity. Additionally, all mathematical rules up to the range of fractional calculus are satisfied by the ABC operators. Numerous academics have discussed the various ABC fractional derivative and fractional derivative notions.

Finding precise answers to fractional differential equations seems to be much more difficult than doing the same for their equivalents with integer orders. The creation of efficient analytical and numerical techniques for approximating solutions to this kind of problem has therefore received a lot of attention. Some of these techniques are the Laplace decomposition technique, Homotopy perturbation method, the Homotopy analysis method and the Adomian decomposition method. Another highly powerful method is the q-homotopy analysis transform approach. This q-HATM fuses the Laplace transform method and the homotopy symmetry analysis method (HAM) when q is in [0, 1/n]. The q-HATM solution converges more quickly than the conventional HAM because it contains the term ${\left(1/n\right)}^{r}$. We solved a system of non-linear equations using the q-HATM in order to find an acceptable remedy for this wild ailment. In order to take into account the new fractional operator known as the ABC operator, the Mittag-Liffler function was contracted with the ABC operator.

## 2 Mathematical optimisation and miniature

The main goal of this investigation is to generalise fractional calculus. We believe that mathematical modelling is a very important element in understanding the disease and its transmission. To manage the amounts of data generated during host-pathogen contact, these types of models must be harmonised. This issue is influenced by a number of variables, including the conceptual analysis of an inhabitant's proliferation of illnesses in susceptible plants and animals. Numerous approaches can be used to analyse the transmission of a disease, and numerous models for pest-tree dynamics have been created [39], [40], [43], [44], [45].

The system of nonlinear equations in this study is subjected to the q-HATM in order to capture the appropriate treatment for the disease. It has been thought that the new fractional operator, known as ABC, produces better outcomes. We evaluated the epidemiology model observing the propagation of the virus in the current study and achieved some results for the epidemic model under consideration. This complex model falls under the category of a non-linear equation. The sensitive pine tree class is represented by the letters M(j), the exposed pine tree class by the letters L(j), the infected pine tree class by the letters N(j), and the susceptible beetle class by the letters O(j). Additionally, P(j) is used to represent the infective class of beetles [34].

At time *j*, we separated the complete population into five compartments: Susceptibility smokers, snuffing class, irregularity smokers, habitual smokers, and quit smokers in this extended model [5], [6]. The model comes from,

(2.1) $$\frac{dM(j)}{dj}=\theta -\chi_{1}M(j)L(j)-\lambda M(j)+\beta O(j)\frac{dL(j)}{dj}=\chi_{1}M(j)L(j)-\chi_{2}L(j)N(j)-(\mu +\lambda )L(j)\frac{dN(j)}{dj}=\chi_{2}L(j)N(j)-(\sigma +\gamma +\lambda )N(j)\frac{dO(j)}{dj}=\gamma N(j)-(\beta +\rho +\lambda )O(j)\frac{dP(j)}{dj}=\rho O(j)-\lambda P(j),$$

where the recruitment rate (birth or migration) is *θ*, the rate of sensitive population moving to snuffing class is $\chi_{1}$, and the rate of snuffing class becoming irregular smokers is $\chi_{2}$. Furthermore, the pace at which nonsmokers become regular smokers is referred to as *γ*, the rate of quitting is *ρ*, the rate of natural death is *λ* and the rate of relapse is *β*. The snuffing class has a high fatality rate due to tobacco use is *μ*, while the mortality rate due to tobacco-related diseases is *σ*.

The associated values of the parametric quantity are $\theta =0.1;\chi_{1}=0.003;\chi_{2}=0.002;\lambda =0.002;\beta =0.003;\mu =0.003;\sigma =0.003;\gamma =0.004;\rho =0.05$ and the initial conditions are, M(0)=68,L(0)=40,N(0)=30,O(0)=20,P(0)=15.

As a result, the ABC derivative model for equation (2.1) of fractional order is presented as follows ([36], [37]).

(2.2) $$\underset{0}{ABC}\;D_{j}^{\chi }M(j)=\theta -\chi_{1}M(j)L(j)-\lambda M(j)+\beta O(j)\underset{0}{ABC}\;D_{j}^{\chi }L(j)=\chi_{1}M(j)L(j)-\chi_{2}L(j)N(j)-(\mu +\lambda )L(j)\underset{0}{ABC}\;D_{j}^{\chi }N(j)=\chi_{2}L(j)N(j)-(\sigma +\gamma +\lambda )N(j)\underset{0}{ABC}\;D_{j}^{\chi }O(j)=\gamma N(j)-(\beta +\rho +\lambda )O(j)\underset{0}{ABC}\;D_{j}^{\chi }P(j)=\rho O(j)-\lambda P(j).$$

Next, we give the important fractional calculus resolutions and theorems, as well as the Laplace transform. Also, we denote K(χ) is a normalising function with K(0)=K(1)=1.

Definition 2.1 For a function $g\in H^{1}(a,b),b>a$, the fractional Atangana-Baleanu-Caputo derivative is defined as follows [8], [9], [16], [37]:

$$\underset{0}{ABC}\;D_{j}^{\chi }(g(j))=\frac{K[\chi ]}{\left(1-\chi \right)}\int_{a}^{j}g^{\prime }(v)L_{\chi }\left[\chi \frac{{\left(j-v\right)}^{\chi }}{\left(\chi -1\right)}\right]dv,$$

Definition 2.2 The fractional Atangana–Baleanu integral equation is classed as:

$$\underset{0}{ABC}N_{j}^{\chi }(g(j))=\frac{\left(1-\chi \right)}{K[\chi ]}g(j)+\frac{\chi }{\left(K[\chi ]\Gamma (\chi )\right)}\int_{a}^{j}g(v){\left(j-v\right)}^{\chi -1}dv,$$

Definition 2.3 The Atangana-Baleanu operator's equivalent, the Laplace transform, is denoted by:

$$L[\underset{0}{ABR}\;D_{j}^{\chi }(g(j))]=\frac{K[\chi ]}{\left(1-\chi \right)}\left(\frac{s^{\chi }L[g(j)]-s^{\chi -1}g(0)}{s^{\chi }+\left(\frac{\chi }{1-\chi }\right)}\right),$$

Theorem 2.1 *The Riemann-Liouville and ABC derivatives, satisfy the following Lipschitz conditions* [7], [17], [18] *:*

$$\left\|\underset{a}{ABC}\;D_{j}^{\chi }g_{1}(j)-\underset{a}{ABC}\;D_{j}^{\chi }g_{2}(j)\|<K_{1}\|g_{1}(x)-g_{2}(x)\right\|$$

*and*

$$\|\underset{a}{ABR}\;D_{j}^{\chi }g_{1}(j)-\underset{a}{ABR}\;D_{j}^{\chi }g_{2}(j)\|<K_{2}\|g_{1}(x)-g_{2}(x)\|.$$

Theorem 2.2 *The solution* $\underset{0}{ABR}\;D_{j}^{\chi }g_{1}(j)=s(j)$ *is given by* *[7]* *:*

$$g(j)=\frac{\left(1-\chi \right)}{K[\chi ]}s(j)+\frac{\chi }{K[\chi ]\Gamma (\chi )}\int_{a}^{j}s(\zeta ){\left(j-\zeta \right)}^{\chi -1}d\zeta ,$$

## 3 The fundamental aspect of q-HATM

To stress the fundamental idea of the recommended method, consider the fractional order nonlinear nonhomogeneous partial differential equation

(3.1) $$\underset{a}{ABC}\;D_{j}^{\chi }\omega (\ell ,j)+R\omega (\ell ,j)+N\omega (\ell ,j)=f(\ell ,j),n-1<\chi <n,$$

where f(ℓ,j) is a concept that has a source, *R* stands for the linear differential operator in *ℓ* and j,N stands for the operator for nonlinear differential equations, and $\underset{a}{ABC}\;D_{j}^{\chi }\omega (\ell ,j)$ denotes the function *ABC* derivative of the function $\omega (x_{i},j)$.

Equation (3.1) has Laplace transform yields the following equation

(3.2) $$\frac{K[\chi ]}{\left(1-\chi \right)}\left(\frac{s^{\chi }L[\omega (\ell ,j)]-s^{\chi -1}\omega (\ell ,0)}{s^{\chi }+\left(\frac{\chi }{1-\chi }\right)}\right)+L[R\omega (\ell ,j)]+L[N\omega (\ell ,j)]=L[f(\ell ,j)].$$

By (3.2), we have

$$L[\omega (\ell ,j)]-\frac{\omega (\ell ,0)}{s}+\frac{1-\chi +\frac{\chi }{s^{\chi }}}{K[\chi ]}L\left\{[R\omega (\ell ,j)]+L[N\omega (\ell ,j)]-L[f(\ell ,j)]\right\}=0.$$

The steps for creating a homotopy for a primary function are as follows:

$$N[\psi (\ell ,j;q)]=L[\psi (\ell ,j;q)]-\frac{\omega (\ell ,0)}{s}\;\;\;\;+\frac{1-\chi +\frac{\chi }{s^{\chi }}}{K[\chi ]}\left\{L[R\psi (\ell ,j;q)]+L[N\psi (\ell ,j;q)]-L[f(\ell ,j)]\right\},$$

where a real function of ℓ,t and *q* is ψ(ℓ,j;q) and q∈[0,1/n].

We construct a homotopy as follows for basic functions with non-zero values:

(1−nq)L{ψ(ℓ,j;q)−ω(ℓ,0)}=hqN{ψ(ℓ,j;q)},

where q∈[0,1/n],(n≥1), *L* is the Laplace transform symbol.

In fact, ψ(ℓ,j;q) is an unspecified function, h≠0 is an auxiliary variable, $\omega_{0}(\ell ,j)$ is an initial estimation of ω(ℓ,j;q).

The following results are acceptable for q=0 and $q=\frac{1}{n}$ respectively:

$$\psi (\ell ,j;0)=\omega_{0}(\ell ,j),\;\;\;\psi (\ell ,j;1/n)=\omega (\ell ,j).$$

The result ψ(ℓ,j;q) converges from $\omega_{0}(\ell ,j)$ to ω(ℓ,j) by expanding *q* from 0 to 1/n.

Using Taylor's theorem near *q* to expand the component ψ(ℓ,j;q) in series structure.

$$\psi (\ell ,j;q)=\omega_{0}(\ell ,j)+\sum_{r=1}^{\infty }\omega_{r}(\ell ,j)q^{r},$$

where

$$\omega_{r}(\ell ,j)=\frac{1}{r!}{\frac{\partial^{r}\psi (\ell ,j;q)}{\partial q^{r}}|}_{q=0}.$$

We have one of the results for equation (3.1), by using the fundamental linear operators $\omega_{0}(\ell ,j),n\&h$.

The series ψ(ℓ,j;q) converges at q=1/n.

$$\omega (\ell ,j)=\omega_{0}(\ell ,j)+\sum_{r=1}^{\infty }\omega_{r}(\ell ,j){\left(\frac{1}{n}\right)}^{r}.$$

By differentiating the zeroth order deformation equation *r*-times with regard to *q*, we are able to compute for q=0 and divide by *r*!

$$L\left[\omega_{r}(\ell ,j)-k_{r}\omega_{r-1}(\ell ,j)\right]=hO_{r}({\overset{\to }{\omega }}_{r-1}),$$

where

$${\overset{\to }{\omega }}_{r}=\left[\omega_{0}(\ell ,j),\omega_{1}(\ell ,j),...,\omega_{r}(\ell ,j)\right].$$

Inverting the Laplace transform of an equation

$$\omega_{r}(\ell ,j)=k_{r}\omega_{r-1}(\ell ,j)+hL^{-1}[O_{r}({\overset{\to }{\omega }}_{r-1})],$$

where

$$O_{r}({\overset{\to }{\omega }}_{r-1})=L[\omega_{r-1}(\ell ,j)]-\left(1-\frac{k_{r}}{n}\right)\left(\frac{\omega (\ell ,0)}{s}-\frac{\left(1-\chi +\frac{\chi }{s^{\chi }}\right)}{K(\chi )}L[f(\ell ,j)]\right)\;\;\;+\frac{1-\chi +\frac{\chi }{s^{\chi }}}{K(\chi )}L[R\omega_{r-1}(\ell ,j)+H_{r-1}],$$

and

$$k_{r}=\left\{ \begin{array}{cc} 0, & \text{if }r\leq 1\\ n, & \text{if }r>1 \end{array}\right.\;H_{r}=\frac{1}{r!}{\left[\frac{\partial^{r}\psi (\ell ,j;q)}{\partial q^{r}}\right]}_{q=0}\;\psi (\ell ,j;q)=\psi_{0}+q\psi_{1}+q^{2}\psi_{2}+....$$

We have,

$$\omega_{r}(\ell ,j)=(k_{r}+h)\omega_{r-1}(\ell ,j)-\left(1-\frac{k_{r}}{n}\right)L^{-1}\left(\sum_{k=0}^{n-1}s^{\chi -k-1}\omega^{\left(k\right)}(\ell ,0)+\frac{1}{s^{\chi }}L[f(\ell ,j)]\right)\;\;\;+hL^{-1}\frac{1}{s^{\chi }}(L[R\omega v_{r-1}(\ell ,j)]+H_{r-1}).$$

Hence, we can obtain $\omega_{r}(\ell ,j)$ iterative term by solving the above. The *q*– HATM's series solution is symbolised by

$$\omega (\ell ,j)=\omega_{0}(\ell ,j)+\sum_{r=1}^{\infty }\omega_{r}(\ell ,j){\left(\frac{1}{n}\right)}^{r}.$$

## 4 q-HATM solution for the prediction phase

We employ the q-HATM solution technique in this section, which is in this section, we use the q-HATM solution technique, which is a classy union of the q- HAM and the Laplace Transform framed by Sing et al. Its benefit is its ability to comprehend powerful computational approaches for perceptive fractional differential equations. Many scientists have improved the technique to discover solutions for other classes of nonlinear differential equations since then. To demonstrate the dynamics in equation (2.2), we employed a fractional-order system of equations

(4.1) $$\underset{0}{ABC}\;D_{j}^{\chi }M(j)=\theta -\chi_{1}M(j)L(j)-\lambda M(j)+\beta O(j)\underset{0}{ABC}\;D_{j}^{\chi }L(j)=\chi_{1}M(j)L(j)-\chi_{2}L(j)N(j)-(\mu +\lambda )L(j)\underset{0}{ABC}\;D_{j}^{\chi }N(j)=\chi_{2}L(j)N(j)-(\sigma +\gamma +\lambda )N(j)\underset{0}{ABC}\;D_{j}^{\chi }O(j)=\gamma N(j)-(\beta +\rho +\lambda )O(j)\underset{0}{ABC}\;D_{j}^{\chi }P(j)=\rho O(j)-\lambda P(j)$$

with initial conditions

(4.2) $$M(0)=M_{0},\;L(0)=L_{0},\;N(0)=J_{0},\;O(0)=O_{0},\;P(0)=P_{0}.$$

We get utilising equation (4.2) as a helper and applying the Laplace Transform to equation (4.1).

$$L\{M(j)\}-\frac{1}{s}M_{0}+\frac{1}{K[\chi ]}\left(1-\chi +\chi /s^{\chi }\right)L\{\theta -\chi_{1}M(j)L(j)-\lambda M(j)+\beta O(j)\}=0\;L\{L(j)\}-\frac{1}{s}L_{0}+\frac{1}{K[\chi ]}\left(1-\chi +\chi /s^{\chi }\right)L\{\chi_{1}M(j)L(j)-\chi_{2}L(j)N(j)-(\mu +\lambda )L(j)\}=0\;L\{N(j)\}-\frac{1}{s}N_{0}+\frac{1}{K[\chi ]}\left(1-\chi +\chi /s^{\chi }\right)L\{\chi_{2}L(j)N(j)-(\sigma +\gamma +\lambda )N(j)\}=0\;L\{O(j)\}-\frac{1}{s}O_{0}+\frac{1}{K[\chi ]}\left(1-\chi +\chi /s^{\chi }\right)L\{\gamma N(j)-(\beta +\rho +\lambda )O(j)\}=0\;L\{P(j)\}-\frac{1}{s}P_{0}+\frac{1}{K[\chi ]}\left(1-\chi +\chi /s^{\chi }\right)L\{\rho O(j)-\lambda P(j)\}=0.$$

The non-linear operator has become projected as follows:

$$N^{1}[\psi_{1},\psi_{2},\psi_{3},\psi_{4}]=L\{\psi_{1}(j;q)\}-\frac{1}{s}M_{0}+\frac{1}{K[\chi ]}\left(1-\chi +\frac{\chi }{s^{\chi }}\right)\;\;\;\times L\{\theta -\chi_{1}\psi_{1}(j;q)\psi_{2}(j;q)-\lambda \psi_{1}(j;q)+\beta \psi_{4}(j;q)\}N^{2}[\psi_{1},\psi_{2},\psi_{3},\psi_{4}]=L\{\psi_{2}(j;q)\}-\frac{1}{s}L_{0}-\frac{1}{K[\chi ]}\left(1-\chi +\frac{\chi }{s^{\chi }}\right)\;\;\;\times L\{\chi_{1}\psi_{1}(j;q)\psi_{2}(j;q)-\chi_{2}\psi_{2}(j;q)\psi_{3}(j;q)-(\mu +\lambda )\psi_{2}(j;q)\}N^{3}[\psi_{1},\psi_{2},\psi_{3},\psi_{4}]=L\{\psi_{3}(j;q)\}-\frac{1}{s}N_{0}-\frac{1}{K[\chi ]}\left(1-\chi +\frac{\chi }{s^{\chi }}\right)\;\;\;\times L\{\chi_{2}\psi_{2}(j;q)\psi_{3}(j;q)-(\sigma +\gamma +\lambda )\psi_{3}(j;q)\}N^{4}[\psi_{1},\psi_{2},\psi_{3},\psi_{4}]=L\{\psi_{4}(j;q)\}-\frac{1}{s}O_{0}-\frac{1}{K[\chi ]}\left(1-\chi +\frac{\chi }{s^{\chi }}\right)\;\;\;\times L\{\gamma \psi_{3}(j;q)-(\beta +\rho +\lambda )\psi_{4}(j;q)\}N^{5}[\psi_{1},\psi_{2},\psi_{3},\psi_{4}]=L\{\psi_{5}(j;q)\}-\frac{1}{s}P_{0}-\frac{1}{K[\chi ]}\left(1-\chi +\frac{\chi }{s^{\chi }}\right)\;\;\;\times L\{\rho \psi_{4}(j;q)-\lambda \psi_{5}(j;q)\}.$$

By stating the provided scheme and for H(x,j)=1, the $r^{th}$ order deformation equation is obtained.

$$L[M_{r}(j)-K_{r}M_{r-1}(j)]=hO_{1,r}[{\overset{\to }{M}}_{r-1},{\overset{\to }{L}}_{r-1},{\overset{\to }{J}}_{r-1},{\overset{\to }{O}}_{r-1},{\overset{\to }{Q}}_{r-1}]L[L_{r}(j)-K_{r}L_{r-1}(j)]=hO_{2,r}[{\overset{\to }{M}}_{r-1},{\overset{\to }{L}}_{r-1},{\overset{\to }{J}}_{r-1},{\overset{\to }{O}}_{r-1},{\overset{\to }{Q}}_{r-1}]L[N_{r}(j)-K_{r}N_{r-1}(j)]=hO_{3,r}[{\overset{\to }{M}}_{r-1},{\overset{\to }{L}}_{r-1},{\overset{\to }{J}}_{r-1},{\overset{\to }{O}}_{r-1},{\overset{\to }{Q}}_{r-1}]L[O_{r}(j)-K_{r}O_{r-1}(j)]=hO_{4,r}[{\overset{\to }{M}}_{r-1},{\overset{\to }{L}}_{r-1},{\overset{\to }{J}}_{r-1},{\overset{\to }{O}}_{r-1},{\overset{\to }{Q}}_{r-1}]L[P_{r}(j)-K_{r}P_{r-1}(j)]=hO_{5,r}[{\overset{\to }{M}}_{r-1},{\overset{\to }{L}}_{r-1},{\overset{\to }{J}}_{r-1},{\overset{\to }{O}}_{r-1},{\overset{\to }{Q}}_{r-1}],$$

where,

$$O_{1,r}[{\overset{\to }{M}}_{r-1},{\overset{\to }{L}}_{r-1},{\overset{\to }{J}}_{r-1},{\overset{\to }{O}}_{r-1},{\overset{\to }{Q}}_{r-1}]=L\{M_{r-1}(j)\}-\left(1-\frac{K_{r}}{n}\right)\frac{M_{0}}{s}+\frac{1}{K[\chi ]}\left(1-\chi +\frac{\chi }{s^{\chi }}\right)\;\;\;\times L\left\{-\chi_{1}\sum_{i=0}^{r-1}M_{i}(j)L_{r-1-i}(j)-\lambda M_{r-1}(j)+\beta O_{r-1}(j)\right\}O_{2,r}[{\overset{\to }{M}}_{r-1},{\overset{\to }{L}}_{r-1},{\overset{\to }{J}}_{r-1},{\overset{\to }{O}}_{r-1},{\overset{\to }{Q}}_{r-1}]=L\{L_{r-1}(j)\}-\left(1-\frac{K_{r}}{n}\right)\frac{L_{0}}{s}+\frac{1}{K[\chi ]}\left(1-\chi +\frac{\chi }{s^{\chi }}\right)\;\;\;\times L\left\{\chi_{1}\sum_{i=0}^{r-1}M_{i}(j)L_{r-1-i}(j)-\chi_{1}\sum_{i=0}^{r-1}L_{i}(j)N_{r-1-i}(j)-(\mu +\lambda )L_{r-1}(j)\right\}O_{3,r}[{\overset{\to }{M}}_{r-1},{\overset{\to }{L}}_{r-1},{\overset{\to }{J}}_{r-1},{\overset{\to }{O}}_{r-1},{\overset{\to }{Q}}_{r-1}]=L\{N_{r-1}(j)\}-\left(1-\frac{K_{r}}{n}\right)\frac{J_{0}}{s}+\frac{1}{K[\chi ]}\left(1-\chi +\frac{\chi }{s^{\chi }}\right)\;\;\;\times L\left\{\chi_{2}\sum_{i=0}^{r-1}L_{i}(j)N_{r-1-i}(j)-(\sigma +\gamma +\lambda )N_{r-1}(j)\right\}O_{4,r}[{\overset{\to }{M}}_{r-1},{\overset{\to }{L}}_{r-1},{\overset{\to }{J}}_{r-1},{\overset{\to }{O}}_{r-1},{\overset{\to }{Q}}_{r-1}]=L\{O_{r-1}(j)\}-\left(1-\frac{K_{r}}{n}\right)\frac{O_{0}}{s}+\frac{1}{K[\chi ]}\left(1-\chi +\frac{\chi }{s^{\chi }}\right)\;\;\;\times L\left\{\gamma N_{r-1}(j)-(\beta +\rho +\lambda )O_{r-1}(j)\right\}O_{5,r}[{\overset{\to }{M}}_{r-1},{\overset{\to }{L}}_{r-1},{\overset{\to }{J}}_{r-1},{\overset{\to }{O}}_{r-1},{\overset{\to }{Q}}_{r-1}]=L\{P_{r-1}(j)\}-\left(1-\frac{K_{r}}{n}\right)\frac{P_{0}}{s}+\frac{1}{K[\chi ]}\left(1-\chi +\frac{\chi }{s^{\chi }}\right)\;\;\;\times L\left\{\rho O_{r-1}(j)-\lambda P_{r-1}(j)\right\}.$$

Using inverse Laplace transforms, we may reduce the preceding equations as follows:

$$M_{r}(j)=K_{r}M_{r-1}(j)+hL^{-1}\{O_{1,r}[{\overset{\to }{M}}_{r-1},{\overset{\to }{L}}_{r-1},{\overset{\to }{J}}_{r-1},{\overset{\to }{O}}_{r-1},{\overset{\to }{Q}}_{r-1}]\}L_{r}(j)=K_{r}L_{r-1}(j)+hL^{-1}\{O_{2,r}[{\overset{\to }{M}}_{r-1},{\overset{\to }{L}}_{r-1},{\overset{\to }{J}}_{r-1},{\overset{\to }{O}}_{r-1},{\overset{\to }{Q}}_{r-1}]\}J_{r}(j)=K_{r}J_{r-1}(j)+hL^{-1}\{O_{3,r}[{\overset{\to }{M}}_{r-1},{\overset{\to }{L}}_{r-1},{\overset{\to }{J}}_{r-1},{\overset{\to }{O}}_{r-1},{\overset{\to }{Q}}_{r-1}]\}O_{r}(j)=K_{r}O_{r-1}(j)+hL^{-1}\{O_{4,r}[{\overset{\to }{M}}_{r-1},{\overset{\to }{L}}_{r-1},{\overset{\to }{J}}_{r-1},{\overset{\to }{O}}_{r-1},{\overset{\to }{Q}}_{r-1}]\}P_{r}(j)=K_{r}P_{r-1}(j)+hL^{-1}\{O_{5,r}[{\overset{\to }{M}}_{r-1},{\overset{\to }{L}}_{r-1},{\overset{\to }{J}}_{r-1},{\overset{\to }{O}}_{r-1},{\overset{\to }{Q}}_{r-1}]\}.$$

Solving the aforementioned equations led us to the equations

$$M_{0}(j)=68\;L_{0}(j)=40\;J_{0}(j)=30\;O_{0}(j)=20\;P_{0}(j)=15\;M_{1}(j)=\frac{8.136h}{K[\chi ]}\left\{1-\chi +\frac{\chi t^{\chi }}{\Gamma (\chi +1)}\right\}L_{1}(j)=-\frac{5.560h}{K[\chi ]}\left\{1-\chi +\frac{\chi t^{\chi }}{\Gamma (\chi +1)}\right\}N_{1}(j)=-\frac{2.130h}{K[\chi ]}\left\{1-\chi +\frac{\chi t^{\chi }}{\Gamma (\chi +1)}\right\}O_{1}(j)=\frac{0.980h}{K[\chi ]}\left\{1-\chi +\frac{\chi t^{\chi }}{\Gamma (\chi +1)}\right\}P_{1}(j)=-\frac{0.970h}{K[\chi ]}\left\{1-\chi +\frac{\chi t^{\chi }}{\Gamma (\chi +1)}\right\}M_{2}(j)=\frac{8.136h(n+h)}{K[\chi ]}\left\{1-\chi +\frac{\chi t^{\chi }}{\Gamma (\chi +1)}\right\}\;\;\;-\frac{0.144588h^{2}}{{\left[K[\chi ]\right]}^{2}}\left\{1-2\chi +\chi^{2}+\frac{2\chi (1-\chi )t^{\chi }}{\Gamma (\chi +1)}+\frac{\chi^{2}t^{2}\chi }{\Gamma (2\chi +1)}\right\}L_{2}(j)=-\frac{5.560h(n+h)}{K[\chi ]}\left\{1-\chi +\frac{\chi t^{\chi }}{\Gamma (\chi +1)}\right\}\;\;\;-\frac{0.373880h^{2}}{{\left[K[\chi ]\right]}^{2}}\left\{1-2\chi +\chi^{2}+\frac{2\chi (1-\chi )t^{\chi }}{\Gamma (\chi +1)}+\frac{\chi^{2}t^{2}\chi }{\Gamma (2\chi +1)}\right\}J_{2}(j)=-\frac{2.130h(n+h)}{K[\chi ]}\left\{1-\chi +\frac{\chi t^{\chi }}{\Gamma (\chi +1)}\right\}\;\;\;+\frac{0.484830h^{2}}{{\left[K[\chi ]\right]}^{2}}\left\{1-2\chi +\chi^{2}+\frac{2\chi (1-\chi )t^{\chi }}{\Gamma (\chi +1)}+\frac{\chi^{2}t^{2}\chi }{\Gamma (2\chi +1)}\right\}O_{2}(j)=\frac{0.980h(n+h)}{K[\chi ]}\left\{1-\chi +\frac{\chi t^{\chi }}{\Gamma (\chi +1)}\right\}\;\;\;+\frac{0.062420h^{2}}{{\left[K[\chi ]\right]}^{2}}\left\{1-2\chi +\chi^{2}+\frac{2\chi (1-\chi )t^{\chi }}{\Gamma (\chi +1)}+\frac{\chi^{2}t^{2}\chi }{\Gamma (2\chi +1)}\right\}P_{2}(j)=-\frac{0.970h(n+h)}{K[\chi ]}\left\{1-\chi +\frac{\chi t^{\chi }}{\Gamma (\chi +1)}\right\}\;\;\;-\frac{0.050940h^{2}}{{\left[K[\chi ]\right]}^{2}}\left\{1-2\chi +\chi^{2}+\frac{2\chi (1-\chi )t^{\chi }}{\Gamma (\chi +1)}+\frac{\chi^{2}t^{2}\chi }{\Gamma (2\chi +1)}\right\}.$$

The given values are obtained by streamlining the above equations. As described by the q -HATM series solution,

$$M(j)=M_{0}(j)+\sum_{r=1}^{\infty }M_{r}(j){\left(\frac{1}{n}\right)}^{r}\;L(j)=L_{0}(j)+\sum_{r=1}^{\infty }L_{r}(j){\left(\frac{1}{n}\right)}^{r}\;N(j)=N_{0}(j)+\sum_{r=1}^{\infty }N_{r}(j){\left(\frac{1}{n}\right)}^{r}\;O(j)=O_{0}(j)+\sum_{r=1}^{\infty }O_{r}(j){\left(\frac{1}{n}\right)}^{r}\;P(j)=P_{0}(j)+\sum_{r=1}^{\infty }P_{r}(j){\left(\frac{1}{n}\right)}^{r}.$$

## 5 Results and discussion

The majority of the problems of the present decade are indeterminable compared to stochastic impact, which offers a practical means of illuminating the viral dynamics. This study looked at the qualitative behaviour of a tobacco snuffing model with a significant number of snuffers, and used the q - HATM to find an appropriate solution to a system of nonlinear computations. In perspective of this paper, the initial conditions for the given scenario are $M(0)=M_{0}=68;L(0)=L_{0}=40;N(0)=N_{0}=30;O(0)=O_{0}=15$; and $P(0)=P_{0}=20$. A series solution was assessed to further understand the model's behaviour. The drawn diagram makes it easier to grasp and comprehend the model. As a result, considering the fractional operator delivers satisfaction in predicting the future model. The diagram demonstrates how the estimated model is significantly influenced by the order and offers greater edibility. We assessed the figures obtained by q - HATM and respectively at time *j* in this segment.

**Figure 1:**
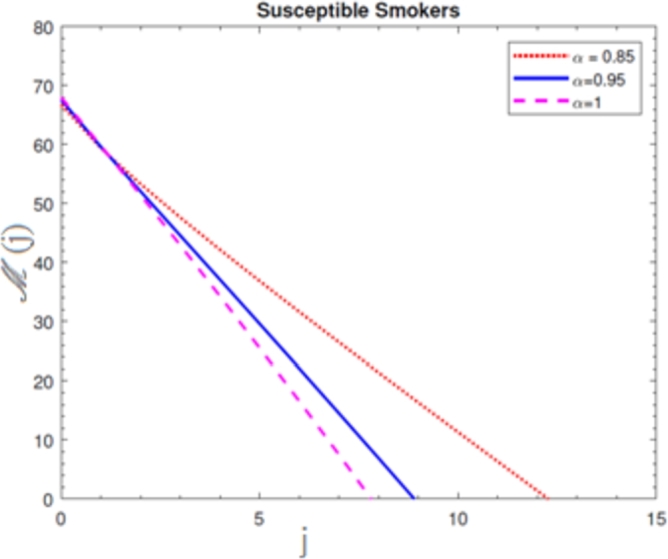
At *h* = −1,*n* = 1, the graph represents a numerical solution for susceptible smokers M(j) over time *j* for various *χ* values.

**Figure 2:**
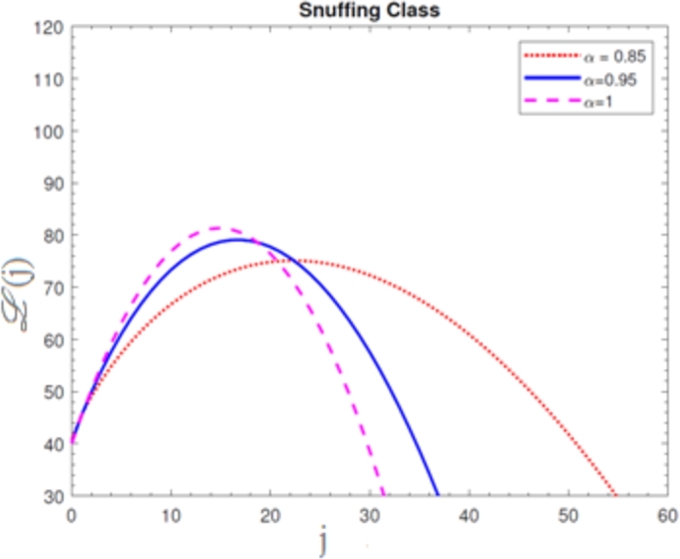
At *h* = −1,*n* = 1, the graph represents a numerical solution for snuffing class smokers L(j) over time *j* for various *χ* values.

**Figure 3:**
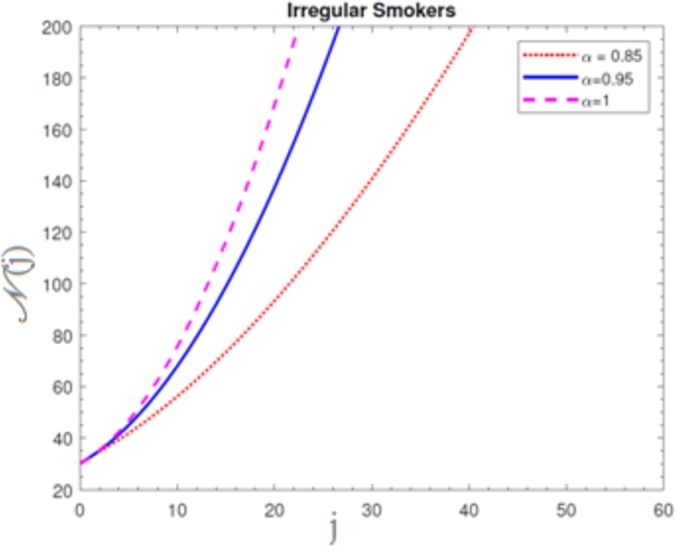
At *h* = −1,*n* = 1, the graph represents a numerical solution for irregular smokers N(j) over time *j* for various *χ* values.

**Figure 4:**
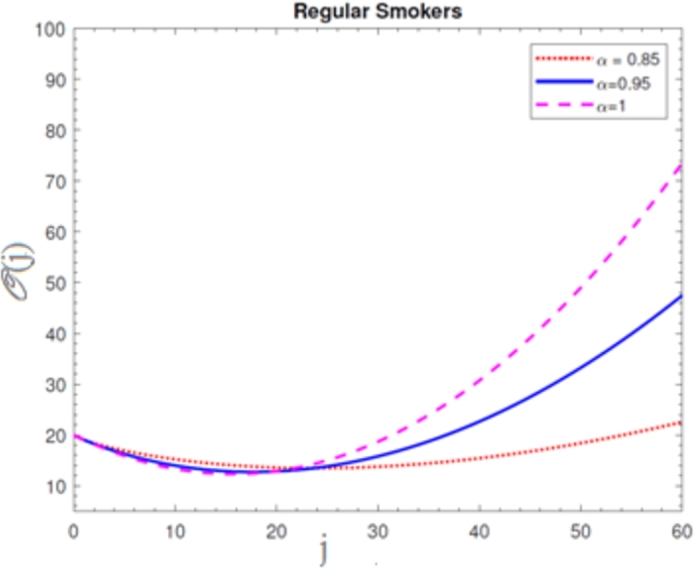
At *h* = −1,*n* = 1, the graph represents a numerical solution for regular smokers O(j) over time *j* for various *χ* values.

**Figure 5:**
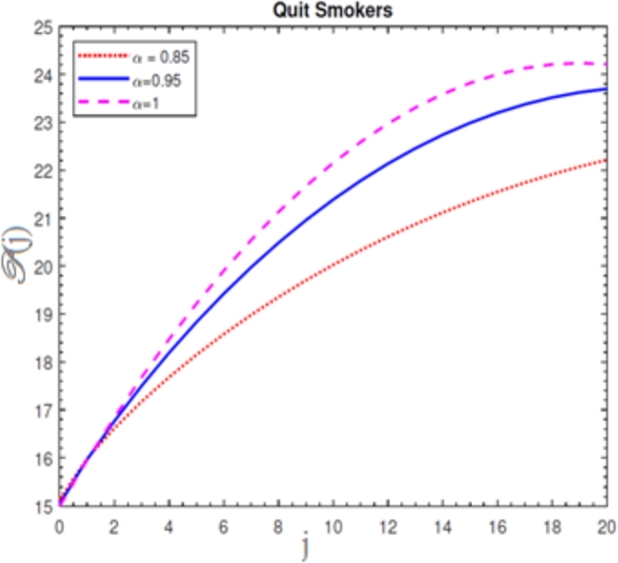
At *h* = −1,*n* = 1, the graph represents a numerical solution for quit smokers P(j) over time *j* for various *χ* values.

A visual representation of the exact simulation for the susceptible smokers M(j) through time *j* for various values of n=1 and h=−1 is shown in Fig. 1. The plotted chart shows that the number of sensitive smokers has decreased dramatically during period *j*. The graphical layout of an accurate simulation for snuffing out class L(j) in time *j* for modified at n=1 and h=−1 is shown in Fig. 2. It can be seen from the graphed data that the snuffing class is declining as time goes on. A graphical representation of the exact simulation for irregular smokers N(j) at time *j* for a range of n=1 and h=−1 values is shown in Fig. 3. From the plotted graph, it can be seen that the number of infectious irregular smokers is rising. Regular smokers O(j) at various fractional orders at n=1 and h=−1 are depicted to demonstrate the unique feature in Fig. 4. The population of frequent smokers is seen to rise for inclined fractional orders. As seen in Fig. 5, the fractional differential co-efficient order at n=1 and h=−1 accelerates the rate of growth for quitters, or P(j).

## 6 Conclusion

In this publication, we have developed and examined a brand-new mathematical model for snuffing and tobacco smoking. Here, the fractional order model of tobacco use with the snuffing class is first formed. We achieved the generalised ABC approach for a numerical solution of the suggested model, which produced outstanding compatibility with results from the q-HATM. The proposed model's graphic results were also shown. Future research could build on the findings to provide cutting-edge mathematical models for smoking and co-infections. The effectiveness of particular smoking cessation methods will be investigated. It should be emphasised that fractional differential equation models require both analytical and numerical approaches. Globally, the Covid-19 pandemic crisis has had an impact on people's economic, social, and mental health conditions. Particularly, anxiety has contributed to a rise in the use of various tobacco products. To recall the past history of smokers in the current model, a current tobacco smoking model with a particular class of tobacco snuffing is transformed into a fractional order in this work. To investigate the model in the form of a fractional order, we employ a fractional derivative in the ABC sense.

## Funding

The work of U.F.-G. was supported by the government of the Basque Country for the ELKARTEK21/10 KK-2021/00014 and ELKARTEK22/85 research programs, respectively.

## Declaration of Competing Interest

The authors declare that they have no known competing financial interests or personal relationships that could have appeared to influence the work reported in this paper.
